# Pyrexia: A Retrospect, a Review, and a Forecast

**Published:** 1885-12

**Authors:** W. H. Spencer

**Affiliations:** Senior Physician to the Bristol Royal Infirmary; Lecturer on Medicine and on Pathology at the Bristol Medical School; President, Bristol Medico-Chirurgical Society, October, 1885


THE BRISTOL

Medico-Chirurgical Journal.

DECEMBER, 1885.

### PYREXIA:
### A RETROSPECT, A REVIEW, AND A FORECAST.

### Inaugural Address

AT THE ANNUAL MEETING OF THE BRISTOL MEDICO-CHIRURGICAL SOCIETY,

BY

W. H. SPENCER, M.A., M.D. CANTAB., F.L.S.,

*Senior Physician to the Bristol Royal Infirmary;*
*Lecturer on Medicine and on Pathology at the Bristol Medical School;*
*President, Bristol Medico-Chirurgical Society, October, 1885.*

GENTLEMEN,—

I have first to thank you very sincerely for the great honour you have done me in calling me to this Presidential Chair. The pleasure and satisfaction which cannot fail to attend the honour conferred would be unalloyed were it not that, in obeying your call, introspection and a reckoning up of deficiencies are involved. Whatever the shortcomings, I rely on your indulgence; and, assured that this will not be withheld, I place such service as I may be able to give unreservedly at your disposal, and assume the high responsibilities of the office to which you have elected me.

The next duty devolving upon me is to read you a Presidential Address. Nor is this, by any means, the lightest of the duties of my office. In selecting a topic upon which to discourse to you, I have been guided entirely by personal considerations. The topic of this address is one that has interested me; the personal interest has determined the selection, and this interest alone. It was selected simply because it happened to be the thing that interested me at the time. For this, perhaps, I ought to offer an apology. But I trust, and I would fain believe, that what has interested me may also interest you; inasmuch as the topic and the point of special interest in it concerns a subject that is always before us as practitioners, and a subject upon which we hunger and thirst, so to say, for fresh knowledge and for newer lights—Fever, to wit.

It was at first my intention to speak of Fever from another point of view. But, as I was busy working up the historical side of my then subject, in that store of old English classics—the Library of the Royal Infirmary—I was drawn to take a different course, because a new interest was awakened. About that time my attention had been directed to the fact that a remarkable application of the mechanical theory of heat to molecular changes, and especially to the interpretation of chemical changes, which had been developing during the past ten years, had made such progress that the new views were commonly taught in our higher schools of chemistry, though they have not yet found their way into the text-books. I refer to the views of chemical combination and disassociation which, discarding the now defunct "affinities" and "repulsions" between atoms, account for chemical change and chemical union on mechanical principles, and deduce the facts of chemical change and chemical union from the law of Dissipation of Energy—itself a deduction from the law of Conservation of Energy.

These new views, developing outside the limits of practical Medicine, seemed to me destined to extend considerably our knowledge of the fever-process. Looking at fever-pathology in the light of this new application of thermo-chemical views, my attention became fixed upon the central and essential fact of Fever—increase of heat with elevation of temperature. I learned that what is to us *the* essential fact, was also accounted *an* essential fact in Fever far back in the history of Medicine. I learned that the accurate observers and acute reasoners of times long past had been struggling to give a due significance and a true explanation to this fact—and, indeed, that they did give such explanations of the fact as the knowledge of the times about the nature of heat and its connections with matter permitted. And I also learned that we are yet a long way off from knowing all the truth about the essential phenomena of Fever—although it may not be presumptuous to exclaim that we see the outline of the goal looming in the distance.

Then, my thoughts took shape in an aphorism: The knowledge of the Present is the outcome of the work of the Past; and the work of the Present is the basis of the knowledge of the Future. In this aphorism the key-note of my address is sounded.

Herein is implied a retrospect; to the better understanding of our present position with regard to the pathology of the fever-process. I invite you to this retrospect. Not for the mere purpose of tracing the development of our knowledge through the observations and reasonings that have led to it; nor, alone, for the sake of an interest that belongs to all history. There is a point of special interest in the retrospect which has not hitherto, so far as I know, been brought into view; and this it is my object to unfold. It will appear, I think, that the notions held in the past as to the pathology of the febrile state have depended upon and progressed with the progression of knowledge concerning the nature of Heat. And the aphorism implies a review; to which also I invite your attention. The knowledge and the work of what we may call the Present—of this present half-century—is in continuity with the knowledge and the work that went before it; and both reflect the notions prevailing in the successive epochs about the nature of Heat. And, further, the continuity of the work and the knowledge of the past with the work and knowledge of the present, must be extended to the future. For, already, the progress of thermo-chemistry has made it clear that the knowledge of the future concerning fever pathology will be the outcome of work now going on, yet only partially developed, in respect of the relations between matter and energy, and energy in the form of Heat. Our aphorism suggests a forecast, to include this future progress; and thus, a welding into a continuous chain of consistent and progressive knowledge the ideas obtained inductively by mechanical and analytical sciences, but drawn off and applied deductively by Medicine in one of her most serious efforts to probe the mysteries of disease. Wherefore, the title of this address.

Before taking up these several points in their order, it will be proper to explain what I mean by the Pyrexial state, and the essential fact of that state. I mean by the Pyrexial state that which *is* fever, and common to *all fevers*. Fever in the abstract, as some would have us believe. "That *abstract* condition which is common to all so-called 'febrile disorders,' and the presence of which gives them their claim to that designation." (Bristowe.) But we must not recognise the use of any such term as "abstract condition" in our practical science, Medicine—if, indeed, the term means anything at all. The Pyrexial state is a fact—a grouping of facts, if you will; it is a state of things that can be experienced and felt: it is open to observation, and its phenomena can be described as matters of fact.

My conception of the Pyrexial state—or, simply, Pyrexia—is this: Pyrexia is a disease-process, set agoing by a great variety of circumstances. It has its own characters, by which we can identify it, and recognise its existence as a factor in many different diseases; in every fever we note certain characters which are repeated, with some variation of detail, in all fevers. These characters define the state. The characters depend on deeper facts, some of which we know: by means of these deeper or essential facts we explain the characters; and by means of these deeper facts the characters of Pyrexia have been generalised in the single fact—elevation of temperature. The deeper facts have also been explained by a large induction and by the application of deductions from chemistry and physics; we know now that the essential fact, the increase of heat (and consequent elevation of temperature), has its origin—at least, in great part—in increased tissue metamorphosis. And this state, Pyrexia, has its own modes of evolution; the characters present definite variations during the working-out of the process. Hence, by study of the evolution of Pyrexia, we are able to construct "types" of fever—as instanced in the hectic and typhoid modes. Whatever we may find in examples of fever more than this—whether characters added, or more explanation needed, or special modes of evolution,—we can always find in these examples the characters, the essential facts and the evolution of the Pyrexial state. In actual examples of a fever, the variations in characters and in details of evolution are apt to be voluminous and complicated; and these are the things that create the interest, and call up the experience, and call out the skill of the practitioner. But in all circumstances and in all vicissitudes, the central and essential fact of Pyrexia remains—viz., increase of heat,—and the characters, modified though they be, remain as witnesses to the presence of that fact.

Now, for the Retrospect. We start from that point which, by common consent, for close upon twenty-four centuries has been held to be the starting-point of positive Medicine—the time of Hippocrates, B.C. 460. It is a common infirmity of the modern medical mind—dazzled, perhaps, by the brilliant lights of modern science—to regard all times anterior to our own as dark and barren of truth, and all medical knowledge and speculation of those times as one gigantic mistake. Most of those who review the history of medical thought and science in times prior to this century, seem rather to be influenced by pity and contempt, and to be actuated by the desire to trace the progress of error instead of the progress of truth. Many facts once supposed to be true, many theories that have caused revolutions in science, have, even after many generations, been refuted, deposed, and dismissed. But many such have also contained something that was sound and permanent. In reading the history of Medicine we must not overlook the truth in our sweeping denunciation of the error in which it may lay embedded.

Hippocrates, as is well known, acknowledged an increase of heat in fevers: he speaks of it as an increase of the *calidum innatum;* and this innate or natural heat of the body he associated with the principle of life. Here is what he says: "What we call heat, that seems to me to be immortal, and to understand, and see, and hear, and know all things whatsoever both present and future." To Hippocrates, this increased *hotness—not heat*—was, as it seems to me, only a symptom; he ventured on no pathology; his business was to observe the superficial characters of disease. He had, indeed, no data on which to found a pathology of fever; he had no theory of heat with which to connect his facts. It required no genius to do what Hippocrates did: to recognise the warmth of the normal living body and the hotness of the fevered body. But he was wise enough to advance no further than his facts and his knowledge allowed; so he said, in effect: What *we call* hotness of the body, whether in health or in disease, is part and parcel of our life.

The influence of the Hippocratic methods and sayings during the succeeding centuries is matter of history. Greek followed Greek along the road that Hippocrates had opened. Great advances in the knowledge of anatomy and disease were made, and the Greeks carried their knowledge into Egypt, where was established the celebrated School of Medicine of Alexandria—a school founded by Ptolemy the First, the brother-in-law of the Macedonian king, Alexander. Then comes the school of the "Methodics," of which school Celsus was one of the brightest ornaments. Celsus, nearly 500 years after Hippocrates, gathered up in his writings all that was Hippocratic in the work of his predecessors. But still there is no trace of a pathology of fever. In Galen's works we first find a trace of a pathology of fever. Galen wrote about 200 years after the Christian era. The præternatural heat of fever was caused, said Galen, by a species of putridity set up in the humours of the body previous to the fever. This conclusion appears to have been reached by connecting the changes observed in stagnant liquids when they are exposed to heat, yet not evaporated, with the appearances in the urine of fevered persons. Here it may be mentioned that this conception of putridity in connection with fever is found in works published in the last century—*e.g.*, Huxham, *On Fever*. Galen also developed the doctrine of "Concoction," a doctrine to be traced in the writings of Hippocrates. This doctrine, applied to fever, asserted that the morbid matters in the humours of the body underwent a change, assisted by the application of heat, which fitted them to be separated and thrown off from the body. This, however, concerns the therapeutics rather than the pathology of fever.

There is no evidence that Galen was an original observer, like Hippocrates; he himself professed only to select and advocate what was true and valuable in the work and opinions of his predecessors. Galen's name and writings are, however, important in the history of Medicine for the influence they had many generations later. Little was done after Galen's time, and up to the revival of learning in the 15th century, to advance the knowledge of Medicine. Here and there a name shines out—*e.g.*, Ætius, Alexander the Thrallian, Paulus Ægineta (7th century), Demetrius, &c.—but what was done seems to have been chiefly in the direction of collecting symptoms and signs of disease, explaining these by theories as to the constitution and changes in the fluids of the body, and devising modes of cure on abstract principles.

During the period when, consequent on intolerance and persecution, men found almost full occupation in guarding and preserving what they could of the knowledge and learning already acquired, we could expect little or no progress in the science, or even in the art of Medicine. As the Dark Ages began to disperse, from Persia and Arabia came back to Europe the medical knowledge and science that then survived; and this was chiefly deposited in the revival-school of Salernum, in Italy. Here the works of the old Greek physicians, which had been translated into Arabic (after the Nestorians, flying from persecution, had settled in Arabia and Edessa in Persia), were re-translated into Latin from the Arabic. And thus Medicine actually begins over again with Hippocrates and Galen fifteen centuries after their time. Cullen says that it was the system and doctrines of Galen the physicians of the 15th century became acquainted with; and that during the 16th century physicians were almost solely employed in explaining and confirming this system and these doctrines. And he accounts for it in this way: that on the revival of learning certain Greek physicians came to Italy, bringing with them their books. The Europeans, studying these books, especially Galen's works, found that they contained almost all the medical knowledge which had been, up to that time, attributed to the Arabians. So the Europeans applied themselves to the study of Galen, as the fountain of medical knowledge, and thus Galenical doctrines became prevalent over Europe. Indeed, down to the middle of the 17th century, when Harvey discovered the circulation of the blood, there was no pathology. Physicians had been busy systematising the superficial appearances and signs of disease; and as to causes, they regarded only those that were proximate. What explanations they gave of the phenomena of disease turned entirely upon the constitution and state of the fluids of the body—a humoral pathology, if the term pathology be applicable at all.

It is surprising that this was so; yet so it was. The notions as to chemistry and natural philosophy had been, up to this time, chiefly applied to therapeutics; although Paracelsus and Van Helmont had suggested explanations of disease—including fever—in accordance with the then chemical doctrines, and the materies morbi of fever was regarded as a mixture of sulphur and saltpetre. But then, this was alchemy, and not chemistry. The iatro-chemical school of physicians had also been founded by Sylvius, a sect who attributed disease to the effects of chemical relations of the fluids.* If we turn to other branches of knowledge, we find the same stagnation that we observe in Medicine during this period. Thus, for Mechanics—a science upon whose progress and development Medicine has depended in no small degree, especially in regard to the nature of heat—from the time of Archimedes, B.C. 212, to the time of Stevinus and Galileo, A.D. 1600 (about), no single advance was made (Whewell).

It comes to this, then, as the result of the first part of our retrospect, from the time of Galen to the 17th century—that, whilst a great store of imperishable "clinical" knowledge was amassed, no progress in pathology was made. Nor was such progress possible, owing to the peculiar circumstances of the times. And in particular, no progress with fever-pathology was made, or was pos-

* But, in truth, chemistry can hardly be said to have existed as a science until the discovery of oxygen by Lavoisier in 1783, though preceded during the 18th century by much good work which paved the way for the further advance.

sible, owing to the imperfect development of those sciences upon which the explanation of the nature of heat depends.

Now comes a period—the 17th century and first half of the 18th century—in which advance in all departments of the physical sciences was rapid and decisive. Francis Bacon introduced the method of induction just at the beginning of the 17th century. Harvey made known the circulation of the blood in 1619, before Bacon died. Galileo propounded the first law of motion in 1638. Descartes and Boyle lived in this century, and Newton followed towards the close of the century. During this century, also, great advances were made in Anatomy and Physiology. The great intellectual activity, and the great and solid advances made in physical science, during the 17th century, were not without effect on the progress of Medicine. And this progress was, as might be expected, on the mechanical side. A few instances may be mentioned. The laws of the motions of fluids came to be applied in pathology. The first conception of heat as motion was introduced. The thermometer was invented in this century. The effect of the discoveries and scientific ideas of this age on Medicine was not only to introduce mechanical notions into Medicine, and to create the iatro-mathematical or iatro-mechanical school, but to induce a more exact method of reasoning upon facts obtained by experiment. Wherefore, the estimation of weight, the calculation of motion, and the measurement of heat, were amongst the results of the work of this age.

Time will not permit a closer scrutiny of this most interesting and instructive period in the history of Inductive Science. Let me therefore illustrate our thesis by reference to the writings of a man who remodelled the

Galenic system in accordance with the mechanical views of the 17th century, and who exerted a great influence upon the further progress of Medicine—Hermann Boerhaave, who lived and taught between 1668 and 1738. And it will be convenient to take him in conjunction with one of his pupils—Van Swieten—who, in his well-known *Commentaries on the Aphorisms of Boerhaave*, gives an insight into the development and practical outcome of Boerhaave's teaching down to the close of the 18th century.

Be it understood, there was as yet no universally accepted scientific conception of the nature of heat. Heat was, for the most part, regarded as a kind of motion in matter; the motion was produced by fire, and fire was caused by a special igneous matter. Bacon, however, said: "Heat is produced by the motion of attrition, *without any preceding heat*." And at the time of Boerhaave it was known that heat caused solids to become fluid. Hooke says: "Heat being nothing else but a very brisk and vehement agitation of all the parts of a body, the parts of a body are thereby made so loose from one another that they easily move any way and become fluid."

Such were the views as to heat prevalent among the natural philosophers of the 17th century. In vol. v. of the *Commentaries on the Aphorisms of Boerhaave*, by Gerald Van Swieten—of an old English edition published in 1757—we find an account of *fever*, as distinct from fevers, which may be thus condensed: The nature of fever, being latent or concealed, must be searched into with great caution. In the search "we are only to consider the appearances of the fever present in the body," to the end that we may understand the proximate cause of fever. But the great number of appearances, or symptoms, attending a fever may lead into error. Wherefore we must select such "symptoms or appearances" as *always* attend *every* fever. And then, from these symptoms, we must thence search into the individual nature of a fever. Boerhaave took *all* the symptoms that had, up to his time, been observed in fevers. He then "blotted out" all those symptoms that occurred only in particular fevers, and retained those which, by the common consent of authors and his own observation, were present in every fever. Then, comparing these, he tried to get at the individual nature of a fever. He was surprised to find three symptoms only as the residue; viz., a shivering, a quick pulse, and heat, varying in degree at different times of the fever. He says "the shivering attends when the fever begins, and is then followed by a heat, gradually increasing, and always the most intense (cæteris paribus) in the height of the fever, decreasing again afterwards by degrees with the fever itself." He then says that the quick pulse is the only symptom present through the whole fever from beginning to end. Therefore, "whatever the physician knows of a fever is derived from the quickness of the pulse." The proximate cause of the velocity or quickness of the pulse is likewise the proximate cause of the fever. Now, this cause is a quicker contraction of the heart, together with increased resistance in the capillary vessels. Elsewhere, in vol. vi. of the same edition of the *Commentaries*, the heat in fevers is discussed separately. The signs by which to distinguish the external heat from the internal heat are first set out. The external heat is to be measured by the thermometer; and rules for using the instrument are given, exactly corresponding to the rules by which we proceed at the present day. The internal heat is known by the redness of the urine.

A very pretty bit of reasoning on the then known facts connects the cause of the increased heat in fevers with the appearances of the urine. But this febrile heat requires a greater quantity of fire; and the greater quantity of fire arises from a more violent attrition of the fluid parts against each other and against the sides of their containing vessels. An objection, raised by one, Schelhammer, is now considered: "The most rapid rivers run swiftly over immense rocks for whole ages together; yet they never become warm, much less do they grow hot;" there must be another principle than motion by which the blood grows hot. Van Swieten answers: Heat may be excited in fluids by attrition, if they are elastic; but otherwise with difficulty. "Yet milk, being swiftly churned, grows warm." Fluids, not elastic, if urged with great force through very small vessels, will grow warm by attrition, especially if the vessels are elastic. Now, Leuwenhoech had already shown that the blood contained a vast quantity of elastic spherical particles—the red globules. In the minute arteries the blood, driven with great swiftness, parts with its thinner portions, which escape through the lateral secretory vessels, and then hardly anything but red globules remains. Thence there comes a violent attrition between these elastic bodies and the sides of an elastic conical vessel, from which attrition heat arises.

The motion of the blood, derived from the increased action of the heart, is then discussed in a philosophical manner in accordance with the laws of motion (so far as then developed). All this is set out in detail with great clearness; even the mechanical alterations in the density of the blood and in the size of the vessels are set forth minutely and explained, with frequent reference to experiment, and the motions are estimated in terms of the law that "the powers in moving bodies are in a ratio compounded of their masses (quantity of matter) and the square of their velocity." Herein is no vain hypothesis, and a twisting of facts to make them support it; but a calm, patient observation of facts—a comparison of the facts with each other and with other facts in outlying sciences, and a reasoning upon the facts after the methods of the Baconian philosophy. Here, indeed, is truth enshrined in error; work of which present knowledge is the outcome; strong, vigorous and exact induction, upon which much of our later deduction rests.

We think those have erred who have supposed that this was a retrograde step in the explanation of the fever-process. On the contrary, it was an important step forwards. It distinctly limited the range of enquiry by applying mechanical principles to the solution of a pathological problem. It *can* be no more than an enquiry into the explanation of symptoms, not—in spite of the word "essence"—an explanation of what we understand as "essential facts." *Signum pathognomicum* are the words used by Van Swieten. Indeed, he says, "There is only this one way of discovering the nature of diseases; viz., by considering each of the *symptoms or appearances* of the disease by itself, then by comparing them with each other, and with what usually happens in a state of health, and from thence, by just reason, to discover the proximate cause of the disease. For thus, by a constant observation, we discover in every fever that the velocity of the pulse is increased, and that therefore the heart contracts more frequently or swiftly; and thence again, that those causes from whence the contraction of the heart results are increased: but in what manner those causes act which excite the heart to a quicker contraction are hitherto concealed from all of us. For all that we know of the nature of a fever, we discover only by its inseparable effects and appearances. Therefore, the three common or inseparable *symptoms* are to be considered in order." Confusion had been made between the *nature*—*i.e.* essential facts—of fever, and the essential, or diagnostically important, *signs* of fever. The old authors, as understood by one another, were not in this confusion at all.

During Boerhaave's lifetime, Frederick Hoffmann, of the University of Halle, had propounded a system of Medicine founded on a theory of nervous influence. This introduction of nerve agency into pathological notions had no real influence on the further progress of pathology, but it gave rise to a deluge of useless systems. Von Haller, following Hoffmann, established a theory of irritability and sensibility, and this led to a long train of neuro-pathological theories of fever. We might dismiss these as wholly unworthy of examination, were it not that some prominent names in the history of Medicine have been associated with the doctrines.

Cullen, who published his *First Lines in the Practice of Physic* in November, 1783—and who spoke with great authority from the Professorial Chair in Edinburgh—adopted and taught a view of the fever-process founded on the theories of Hoffmann and Haller. He says: "The idea of fever, then, may be, that a spasm of the extreme vessels, however induced, proves an irritation to the heart and arteries (whereby their action is increased), and that this continues until the spasm is relaxed or overcome." The spasm he conceived to be a part of the operation of the *vis medicatrix naturæ*.

The view we are inclined to take of these doctrines is this: that the physiology of the nervous system is such in its nature as to provoke to great speculative application; and when this subject broke fresh in upon the Medicine of that age, it found men in that temper to indulge in unlimited speculation. Chemistry was at a standstill; mechanics had been applied until men had gone as far as they could with it. What wonder that the mysterious and subtle phenomena of nervous action and electricity should have been eagerly seized upon as containing the true key to all medical problems! The attempt was even made to explain fever by an application of the doctrine of polarity!

It was reserved for Chemistry to make the next great step in advance.

Towards the close of the 18th century, the work of Black, and Cavendish (who first decomposed water), and Priestley and Lavoisier, made a fresh and most important epoch in the history of chemistry. Priestley and Lavoisier separately discovered oxygen in 1783. This discovery soon gave birth to the theory of combustion known as *oxidation*. Lavoisier, together with Laplace, made enquiries into the causes of animal heat; and carrying the principle of oxidation into their researches, attributed animal heat to the union of oxygen with hydrogen and carbon in the lungs during respiration. Lavoisier thus sums up his conclusions: The animal machine has three regulators—*respiration*, by which hydrogen and carbon are consumed and heat is produced; *transpiration*, by which temperature is lowered and the body is cooled; *digestion*, by which the blood regains what it had lost. Whilst this continued to be the generally accepted doctrine of animal heat down to about the year 1840, neuro-pathological theories were still applied to the explanation of fever heat, except in France. Even in 1812 Sir Benj. Brodie adduced experimental evidence (in a paper published in the *Philosophical Transactions*) against the theory of the production of heat by the respiratory processes, and concluded that the source of heat must be sought for in the nervous system. And this was nearly fifty years before the celebrated experiment of Claude Bernard—division of the cervical cord of the sympathetic. Meanwhile, chemists and physicists were making rapid strides towards that great generalisation of modern times—the law of the conservation of energy. The time when that law was finally announced marks the end of our retrospect. During the period now under consideration—*i.e.*, from the middle of the 18th century to about the year 1850—great changes occurred and great advances were made in the views as to the nature of heat. The development of the dynamical theory of heat preceded, and was the ground of, those researches on which are based our present views of fever-pathology.

In 1757, Black established the doctrine of Latent Heat, having previously discovered carbonic acid gas, or, as Black called it, "fixed air." The analogy between the fixed and the free states of carbonic acid gas—fixed, *e.g.*, in marble, free when the marble is acted upon by an acid, —and the sensible and latent states of heat—fixed or latent in steam, free or sensible in the water condensed from steam,—led to the conception of heat as a *substance;* a substance which took part in the constitution of matter. But, since gases were found to have weight, whereas the presence of heat in a body could not be detected by the balance, the substance, heat, was called "an imponderable substance." And electricity, light, and even the so-called vital force, were included in the list of "imponderables."

Count Romford, at the end of the last century, by his well-known and beautiful experimental investigations, disproved the materialistic theory of heat. There is this in Romford's work and results which is of special interest to us: he saw clearly that the force of animals was derived from their food, and that no *creation* of force took place in the animal body. Davey followed Romford, and taking the materialists on their own ground, upset them completely. The sum of the results of these observers may be thus expressed: that when work is spent in overcoming friction, the amount of heat produced is proportioned to the work spent. Then came the expression of this relation numerically. The experiments and measurements of Dr. Joule, of Manchester, established in 1843 the mechanical equivalent of heat. At this time, a member of our own profession, a practising surgeon at Heilbronn, in Germany—Dr. J. R. Mayer—was busy formulating his doctrine of the unity and co-relation of all forces—whether mechanical, chemical, thermal, or electrical. He appears to have arrived at this notion of the interdependence of forces from his reflections on the relations between the thermal, chemical, and mechanical phenomena of the body and the force contained in food. Mayer worked out theoretically and enunciated the convertibility and indestructibility of force. Joule contributed the experimental basis upon which the doctrine rests as a law of science. The law was, at this time, called the Law of Conservation of Force.

Heat, up to this time, was matter; the view accepted now is the antithesis: Heat is a mode of motion—translated mechanical motion. The idea of heat as a *vibratory*

motion of the particles of a body had not yet taken deep root—although individual thinkers (notably Newton and Cavendish) had, for a long time before, conceived even this state of things in reference to heat.

Meanwhile, the progress of organic chemistry had been great. A connection between the constituents of food, the heat of the body, and the chemical processes going on in the *tissues* of the body was established. Accordingly, the facts of organic or animal chemistry, together with the doctrines of the Dynamical theory of Heat, were the bases of the researches of Parkes, Senator, Liebermeister, Virchow, Burdon Sanderson, and others; the foundation upon which was elaborated most of our present knowledge of the Pyrexial state.

It is now some ten years since any noteworthy addition was made to our knowledge of the nature of Fever. It is as if the oars rested after so desperate and brilliant a struggle over so long a course. The knowledge acquired by the men just named is our present knowledge. If, then, we recount our present knowledge of the pathology of the Pyrexial state, we shall at once complete our retrospect of the work of the past, and enter upon a review of the knowledge of the present. But, not only is the time too short for such an exposition; this knowledge must be familiar to us all. Wherefore, a brief summary must suffice; and our review shall be chiefly of the work of the present in chemistry and physics, as a prelude to the forecast which is to follow. Of the *work* of Parkes and his worthy contemporaries it will be enough to say, that the experiment was laborious and the observations were accurate, and both were perfectly adapted to secure the ends held in view; that the conclusions arrived at were obtained by just and scientific reasoning such as Bacon and Boerhaave might have taken for their model. Of the knowledge which came out of it all, here is the epitome. Premising that the elevation of temperature is due to increased production of heat, it was established:

1. That the nitrogen discharge of health is in direct proportion to the nitrogen ingestion.
2. That a man in fever discharges more nitrogen than a man in health—both being on the same nitrogenous diet.
3. That the excess of nitrogen = $\frac{3}{4}$ of the normal discharge—*i.e.* the nitrogen excess in fever is more by $\frac{3}{4}$ of the normal discharge. (Unruh.)
4. That, in fever, there is an excess of discharge of carbonic acid which is not accounted for by increased respiration.
5. That the amount of potassium salts discharged in fever is three or four times greater than the amount discharged in non-febrile states. There is also an increased discharge of pigmentary matters in fever about equal in excess to the potassium salts. (Salkowski.)

How are these facts related?

In health, all the nitrogen discharge is derived from the food.

When there is practically no nitrogen income, as in fever, the nitrogen discharged (in excess) must come either from the circulating or the fixed albumen.

Now, in fever the nitrogen income is as defective as it is in inanition.

The circulating albumen is used up in fever. But, besides this, there is an additional disintegration of albumen which, from the fact of the increase in the discharge of potassium salts, probably takes place at the expense of blood corpuscles and muscle, or other tissue.

Wherefore, the tissue-origin of the increased production of heat in fever is now an accepted fact.

That there is an intimate relation between the states and activities of the nervous system as a whole, or of some of its parts, and the maintenance of fever heat there can also be no question. At present, however, this subject has not come within the range of experimental investigation.

We pass on, then, to inquire in what way physics and chemistry have carried on the work of the past, in this half-century, and what physics and chemistry are now doing towards a basis for the knowledge of the future.

And first we note a change in nomenclature, and a change in the forms of thought, for both sciences, from the time we last spoke of them—at the epoch of the enunciation of the law of Conservation of Force. Nor is this change confined to physics and chemistry: all other departments of knowledge that depend upon these sciences—and what departments do not?—must needs run with the stream. Physiology is now fully one with physics and chemistry in this respect.

The change is not a mere change of names for things. The change may be traced—through steps which need not now be detailed—to a wider and more exact conception, developed during the near past and the present, of the law of the Conservation of Force, and to wider applications of the principle. The law as it now stands is called the law of the Conservation of *Energy*. As at present understood, its speciality, as it were, consists in the comprehensive and accurately scientific idea expressed by the word Energy. As we now define it, energy is the capacity for doing work. But energy is, after all, only a new name for a *quantity*, which came before us first during our retrospect of the mechanical work of the 17th century; an old conception, widened with the progress of science. It is essentially the old quantity by which the power of a moving body was expressed in the time of Galileo—the product of the mass into the square of the velocity; the *vis viva*, a term used by Liebnitz later on to express the same thing; the Kinetic energy of to-day. [$F = \frac{1}{2} M V^2$.] Heat is now regarded as one of the forms of energy; and the energy is believed to exist in a hot body in the form of rapid and irregular, though limited, motions of the invisible particles of the body—as Kinetic Energy. Energy may be measured in the form of heat; and every kind of energy can be converted into heat. All we know about heat is what occurs when it passes from one body to another, and we must be careful not to assume that when energy in the form of heat enters a substance, it exists in that substance as heat. The old notion of "latent heat" is exploded, and a new meaning has been given to that term. By the teaching of modern physics, all that we know about matter relates to the series of phenomena in which energy is transferred from one portion of matter to another; we know matter only as that which may have energy communicated to it from other matter, and which may, in its turn, communicate energy to other matter. All that we know about energy is relative; we can ascertain the increase or diminution of energy, as a quantity, as any material system passes from one definite condition to another. There is no such thing as absolute energy; and even if there were such a thing, knowledge of the fact would be of no use to us, since all phenomena depend on the variations of the energy. These newer ideas and ways of physics have found their way into the thoughts and work of physiologists for more than these ten years past. And much has come out of it. For new knowledge in regard to the development and disposal of heat in the body has been gained, and still the newer kind of work is going on; so that in a little time much new knowledge may be available for use to the progress of Medicine.

Within recent years, a most important deduction from the law of Conservation of Energy has been made—a deductive principle we owe to Sir W. Thomson, and known as the law of the Dissipation of Energy. This law affirms (what also belongs to the Conservation of Energy) that the quantity of energy in the universe is invariable, and that energy can neither be created nor destroyed. But it goes on to affirm that every change in the form or distribution of energy is attended, on the whole, with a running down of energy—that is, a diminution in the amount of work it can be made to do. This is also expressed by the phrase "loss of potentiality." Thus, if mechanical energy be converted into heat, this heat cannot be entirely converted back again into work; and this is owing to the diffusive nature of heat. So that the result of continually converting mechanical motion into heat will be that the amount of work to be got out of the heat will be always growing less, until all the energy has taken the unavailable form of diffused heat.

That the further development and application of this law is pregnant with great consequences to physiology and pathology cannot admit of doubt. Already, by the help of this law, the chemist of to-day is busy seeking new explanations of the phenomenon of chemical combination. In the present, not only is chemistry gaining a great store of new facts as to the constitution and changes of organic bodies; the work in thermo-chemistry now going on must produce far-reaching results. And it is, in my opinion, here that we must look for help in the further advancement of fever-pathology.

It was, for a long time, the prerogative of chemists to be concerned with the smallest and ultimate particles of matter. Physics stepped in and usurped authority in this domain. And physics, ever since, has maintained the lead. But now chemists, bowing to the inevitable, are following the lead of the physicists, and are applying mechanical notions to the solution of chemical problems. The principle of the Dissipation of Energy is being applied to explain the reason why elements arrange themselves in certain groups to form chemical compounds. Vibration in unison, harmony of motion of atoms, consequent on the tendency in nature towards an equal distribution of energy, may, it is seriously suggested, be the cause of equilibrium amongst atoms and the forming of chemical compounds—without need of invoking a special kind of attraction, called "chemical affinity." The principle of the equivalence between mechanical work and heat has been extended so as to embrace all molecular actions; as, for instance, the motion of molecules during change of state, and during chemical changes. The principle of the mechanical equivalent of heat being supposed to be true for molecules as well as for sensible masses, its application to molecular work has led to consequences which experiment confirms in a manner remarkably constant. The agreement between the theoretical deductions and the experimental verifications permits the application to all chemical phenomena, with something very like certainty, of that relationship which exists—according to physics—between heat lost and work done. And thus the celebrated French chemist, Berthelot, deduces from the law of Conservation of Energy his principle—the principle of the heat equivalence of chemical transformations.

It is almost startling when one realises what this new development of mechanico-chemistry means. Thus, in respect of chemical combination, molecules are precipitated upon one another with great velocity, and a disengagement of heat results. Each of the invisible molecular masses is the seat of different kinds of motion —motion of translation, of rotation, of vibration,—all motions which are ordinarily destroyed or transformed in the formation of a new compound. The distances between the molecules are changed, and consequently their reciprocal actions; new relations and conditions are evolved. The work done during all these various changes is expressed, just as in the case of sensible masses, by the heat disengaged. The mind needs to become accustomed before it can realise that things so subtle and delicate are now, in this present, the subjects of calculation in terms of *vis viva* and of experimental verification; still more, before it can realise the enormous influence which such researches must exert on the progress of science, and through this on the progress of Medicine.

Berthelot carried his investigations into the domain of physiology, and established new facts in regard to the production of animal heat—or, at least, has given to certain facts a new importance. Here, we can only indicate and suggest, and that briefly, how these things may bear upon the knowledge of the febrile state of the future.

Here is the heat. What of the work out of which it comes?

Berthelot's first theorem, formulated as a basis for the calculation of animal heat, runs thus: "The heat developed by a living body, during any period of its existence, accomplished without the aid of any outside energy except what is derived from its food (oxygen and water included), is equal to the heat produced by the chemical metamorphoses of the immediate principles of its tissues and its food, diminished by the heat absorbed in the external work done by the living body."

The development (as we term it) of heat in the body is not an origination or production *de novo;* it is an alteration in the distribution and dissipation of energy. And this redistribution of energy is brought about chiefly by changes of a chemical nature. Now, these chemical changes are of very diverse kinds—oxidations, hydratations, de-hydratations, decompositions. Each of these reactions disengages or absorbs heat, and each in its own way and on its own account.

Hitherto, in the investigation of fever, attention has been directed almost exclusively to oxidations. But we must not pin ourselves down to the idea that, when we have established metamorphosis of particular tissues, and by oxidation, as a source of the heat in fever, we have set a limit to the source of fever-heat. Berthelot and others have found that, judged from the new point of view—the calculation of the *vis viva* of molecular energies,—the influence of the other changes above-mentioned in the production of heat must be very great. For instance, decomposition is a source of heat quite independent of all oxidation. Hydrates of carbon, sugars and their analogues, disengage heat by decomposition alone, independently of oxidation. Fatty bodies can give rise to heat in the same manner, and also by simple hydratation. The albuminoid principles are amides, and, like amides, give rise to acute heat phenomena at the time of their hydratation with decomposition, or their de-hydratation with combination. And these phenomena occur without the absorption of any oxygen or the production of any carbonic acid.

These points have been merely glanced at. But the facts referred to, and others like them, will have to be taken into account in further researches as to the source of fever-heat.

One word must be said here as to the participation of the nervous system in the production of febrile heat. Hitherto nervous agency has been chiefly invoked in explanation of heat regulation; though recently an interaction between the nervous system and blood and tissues has been inferred in the production of heat in fever. *Tension in nerve centres* is a favorite expression of the day. But what *is* nerve-tension? Does anyone apprehend it? Can it be expressed as a state of things in which molecular motion and energy are concerned? If so, it may have some connection with fever-heat. It might be asked if nerve-tension is a normal or a diseased state; and if it is a diseased state, whether it is a cause or an effect of the febrile heat.

Our knowledge of the Pyrexial state is no doubt great; and true, so far as it goes. Yet are we only on the threshold of the truth. For, as always happens in science, the problem gets more and more complicated in proportion as we penetrate into the actual conditions of natural phenomena.

It was discovered by Wanklyn, some years ago, that sodium heated in dry chlorine gas was not acted upon by the chlorine; no chloride of sodium could be found, and the sodium remained perfectly bright and metallic. Harold Dickson (of Oxford) has recently shown that carbonic oxide and oxygen, when mixed in the proper proportions, will *not* combine to form carbonic acid, even by means of an electric spark, *if* the gases have been thoroughly dried. No doubt, we have, most of us, performed the simple experiment of placing sodium in an atmosphere of chlorine, and have seen the sodium burn even without being heated; and therefore we have believed the fact. It *was* thought that we *knew* how sodium is acted upon by chlorine. *Now* we find we do not know, because, under certain conditions—the almost total absence of moisture,—chlorine will not act on sodium at all. And we don't know *why*.

It *was* a fact in astronomy that the earth and the planets move in ellipses round the sun. This is, however, only an approximately correct statement. The more the actual natural phenomena of astronomy have been penetrated, the more it has been found that the truth about the motions of the earth and planets is inconsistent with the original *simple* explanation. And so it is in all Nature.

> "Knowledge grows, and thro' the ages one increasing purpose runs,
> And the thoughts of men are widen'd with the process of the suns."

